# Energy-Efficient Packet Forwarding Scheme Based on Fuzzy Decision-Making in Underwater Sensor Networks

**DOI:** 10.3390/s21134368

**Published:** 2021-06-25

**Authors:** Jitander Kumar Pabani, Miguel-Ángel Luque-Nieto, Waheeduddin Hyder, Pablo Otero

**Affiliations:** 1 Institute of Oceanic Engineering Research, University of Malaga, 29010 Málaga, Spain; jitander.pabani@uma.es (J.K.P.); pablo.otero@uma.es (P.O.); 2 Department of Telecommunication Engineering, Dawood University of Engineering and Technology, Karachi 74800, Pakistan; 3 Department of Computer Science, Faculty of Science and Technology, Ilma University, Karachi 75190, Pakistan; fhyder152@gmail.com

**Keywords:** underwater sensor networks, packet forwarding probability, fuzzy logic

## Abstract

Underwater Wireless Sensor Networks (UWSNs) are subjected to a multitude of real-life challenges. Maintaining adequate power consumption is one of the critical ones, for obvious reasons. This includes proper energy consumption due to nodes close to and far from the sink node (gateway), which affect the overall energy efficiency of the system. These wireless sensors gather and route the data to the onshore base station through the gateway at the sea surface. However, finding an optimum and efficient path from the source node to the gateway is a challenging task. The common reasons for the loss of energy in existing routing protocols for underwater are (1) a node shut down due to battery drainage, (2) packet loss or packet collision which causes re-transmission and hence affects the performance of the system, and (3) inappropriate selection of sensor node for forwarding data. To address these issues, an energy efficient packet forwarding scheme using fuzzy logic is proposed in this work. The proposed protocol uses three metrics: number of hops to reach the gateway node, number of neighbors (in the transmission range of a node) and the distance (or its equivalent received signal strength indicator, RSSI) in a 3D UWSN architecture. In addition, the performance of the system is also tested with adaptive and non-adaptive transmission ranges and scalable number of nodes to see the impact on energy consumption and number of hops. Simulation results show that the proposed protocol performs better than other existing techniques or in terms of parameters used in this scheme.

## 1. Introduction

An Underwater Wireless Sensor Network (UWSN) is a collection of sensor nodes, which together perform collaborating tasks to measure environmental conditions including temperature, pressure, salinity, turbidity, water quality, absorption loss due to frequency at specific depths at various levels under the sea, oceans, river, and lakes. Besides that, their applications extend to seismic monitoring, underwater mine searching, submarine tracking, pollution monitoring in oil and gas pipelines, etc. A distributed UWSN can give a quick and not expensive solution to a fast deployment for measuring parameters that can be dangerous for the sea environment, e.g., determining the volume and direction of the oil or fuel leak, evaluate the density and direction by effect of marine currents of banks of toxic algae, and so on. In addition to monitoring and performing certain tasks in shallow and deep water underwater communication, underwater communications suffer from undesired effects, like bottlenecks due to long propagation delays, limited available bandwidth at lower frequencies or high absorption of acoustic energy at frequencies of hundreds of kHz, and noise from different sources. The UWSNs look very different when compared with Terrestrial Wireless Sensor Networks (TWSNs). UWSNs [1] consist of sensor nodes which are deployed at locations that may have different depths in the sea volume. These sensor nodes collect the data at their locations and send the data to the sink node (sometimes called gateway node or simply gateway) at the sea surface which sends the data to the on-shore data gathering station. On the contrary, a TWSN uses radio frequency as communication medium and employs traditional protocols like Transmission Control Protocol (TCP), which are not suitable in the underwater environment due to many reasons including attenuation and shorter range. The common classification of sensor nodes is related to their mobility: mobiles, statics, and hybrids. In the mobile category, the sensor nodes are free to move or Mobile Ad hoc Network (MANET) (they are not anchored to the seafloor), which might cause changes in the network topology. It is the case of Autonomous Underwater Vehicles (AUVs) or sea gliders. The static nodes mean that nodes cannot move freely. In a 2D setup architecture in UWSN the static sensors are generally placed near the sea surface and also communicate with the gateway. In a 3D architecture, the nodes are be deployed at different depths using the appropriate gadgets anchored to the seabed. When the gadgets are wire based, the nodes will move around a central position due to the sea currents, which makes the design of a routing protocol more challenging than for a static 3D network. The hybrid setup, which employs a combination of the mobile and static sensors, is used to accomplish specific tasks [2].

Underwater networks vastly suffer from major factors that can affect their overall performance. These factors include channel utilization, localization, routing issues, choice of optimal packet size, environmental effects, MAC issues, etc. The behavior of the underwater acoustic communication channel (UAC) is affected by multiple factors like multipath fading and the Doppler effect, which is due to movement of both sender and receiver nodes, low and variable acoustic propagation velocity, and noise. To cope with all such issues, routing protocols for UWSNs play a vital role and, thus, routing is a primary concern. Generally, routing protocols are categorized into two main classes: proactive and reactive. The proactive routing is a table-driven routing protocol. All nodes store information of each route in the network and network topology changes as the changes in the status of the network occur. This results in low latency because nodes already know where a packet must be forwarded based on routing information stored, hence the forwarding delay is short. On the other hand, the reactive routing protocols find the route on demand by sending many request packets, which results in huge acoustic delays because of the low speed of sound, and this makes the reactive protocols not appropriate for UWSNs [3].

One of the most important undesired effect in UWSNs, is the node death due to improper consumption of energy which is usually caused by inappropriate or inefficient selection of routing. Thus, to address this, the need of an energy efficient routing protocols is of extreme necessity. The main criteria to choose a routing protocol are based on how to select the forwarding node. This node selection process depends on several factors such as distance, number of hops, residual energy, etc.

In this work, the problem of energy efficiency in routing protocols in underwater acoustic communication networks is addressed. A routing algorithm is proposed that improves the energy efficiency of a previous work [4]. The principle of operation of the new algorithm chooses the forwarding node by fuzzy logic interference. As the unknown structure of the network topology, a wide set of rules has been envisaged and implemented inside the core of the fuzzy decision. The results will demonstrate and quantify the pursued objective: decreasing in the average energy consumption of the network.

The paper is structured in six different sections. Section 2 contains the state-of-the-art. Section 3 describes the system model considering network 3D model, propagation, and energy consumption model. Section 4 explains detailed operation of proposed protocol, followed by Section 5 in which performance results are presented. Finally, a detailed discussion on the purpose and performance of the proposed protocol is found in Section 6.

## 2. Related Work

The main characteristics of the network considered in this work are random topology, multi-hop relay, and single gateway. Regarding the second one, the work in [5] proposes multi hop transmission protocol in which the packets are sent from one hop to another hop and finally to the sink. The packets from source node to sink are selected via cluster head using fuzzy logic. This approach uses three input parameters, current energy, trust factor, and distance from base station are calculated to the selected cluster. If the cluster head has a greater number of cluster heads between sink and itself, it employs the fuzzy logic to choose the preferred cluster head to reach the data. The neighbor node, nearer to sink can be elected by the best forwarding node. The best forwarding node is selected based on high trust factor and distance to sink node. The protocol results in increased lifetime and reduces overhead in the network.

A multi-hop network is also considered in [6]. These authors propose a location-free single copy protocol (RECRP); no extra hardware is needed to determine the location, parameters like received signal strength indicator (RSSI) and Doppler scale shift measurement (relative speed) are used for estimation of distance. Transmission power and channel frequency parameters are dynamically controlled by optimal min-max technique. It uses two-hop forwarding capabilities to achieve energy efficiency. Another benefit of the protocol is that it can prevent communication voids. The protocol results in decreased energy per node per message and end-to-end delay compared to other techniques, while keeping an increased packet delivery ratio.

The energy efficiency issue has been addressed in SOSRP [7], where a decentralized self-organizable scalable routing protocol is proposed, where a node failure does not affect the communication in the network. It is a hop-by-hop-based communication protocol where messages are forwarded to the gateway by the relay nodes. The scenario considers nodes deployed randomly at different depths. Initialization of the network is done by means of HELLO packets; the gateway broadcasts this packet among nodes within its transmission range. After the packet is received, a node increments a counter, stores the hop count and re-broadcasts the packet. A temporary failure is introduced to further test the system and identify fault tolerance. This scheme is based on 3D distance between source and destination and hop count. The work in [8] also addresses the energy efficiency issue. The routing protocol EECOR (Energy Efficient Cooperative Opportunistic Routing) is proposed to forward the packets towards the sink. The source node determines the forwarding relay set based on local information of the forwarding node. To select the best relay, fuzzy logic is used considering two input parameters, i.e., energy consumption ratio and packet delivery probability. The output value is described by a figure of merit called Chance; a high value of Chance in the proposed scheme indicates that the neighbor node in the forwarding relay has the opportunity to be selected as best relay. In addition, to avoid packet collision, holding time is introduced for each forwarder to schedule packet transmission towards the sink. The protocol results in better end-to-end delay due to avoidance of packet collision and achieves lower energy consumption. However, the drawback is that parameters such as distance, transmission range, and hop count are not considered while designing the protocol. Fuzzy logic is used in this work to improve energy efficiency. Nevertheless, fuzzy logic had been previously used in [9] BHUSHAN for parent node selection and also for scheduling and tree formation. The selection of the forwarding node is made based on the minimum number of dynamic neighbors.

Another common approach is clustering, also used in a work already presented [5]. In FBECS [10], the protocol is cluster based in which sensor nodes send or forward the data to their respective cluster heads. In this scheme, an eligibility index is calculated for each node for selecting the appropriate cluster head. The parameters considered for eligibility are remaining energy, distance from the sink, and node density. This protocol achieves load balancing by selecting the best candidate to be the cluster head based on the parameters considered. A problem that usually appears in these cases is that a node is isolated, that is, it does not belong to any cluster. In [11], the authors propose a solution for forwarding node selection that usually causes energy imbalance in the network and creates the void hole, which is the situation when a node has no next hop forwarding node in its transmission range, and due to this void node, the data forwarding stops. To prevent void holes, the preferred forwarding nodes are selected inside small cubes to reduce interference and making routing decisions more efficient, which results in enhanced lifetime of the network and also packet delivery ratio. Besides that, a three-dimensional division of the network is done which makes the network scalable, and linear programming is used to reduce end-to-end delay and energy consumption and to increase packet delivery ratio in the network.

Another approach to choose the forwarding node is the location-based protocol. The Relative Distance-Based Forwarding (RDBF) routing protocol [12] is a protocol based on this scheme. A fitness factor is used to select the appropriate node, which reserves the right to forward packets to nodes whose fitness factor is better than a threshold; only those nodes will participate in the forwarding process. Thus, the benefited relay nodes are selected based on shortest distance from the gateway and minimum hop count to forward the packets. Therefore, only a small number of nodes are part of the forwarding process, which reduces energy consumption and also reduce the end-to-end delay. It also selects the optimal path from source nodes to the gateway, in terms of residual energy and distance. This RDBF protocol also has the advantage of controlling transmission time for multiple forwarding nodes, which helps in reducing the duplication of packets. Perhaps the simplest technique to choose the forwarding node is to consider the number of hops. A priority function is introduced in the RPSOR (Reliable Path Selection and Opportunistic Routing) protocol [13] for UWSNs to select the forwarding packet and to choose the nodes that need the smaller number of hops to reach the gateway. This is done through the Shortest Path Index (SPI) parameter for every node that forwards data. In addition, the parameters considered for calculating the SPI are hop count, weighting depth difference sum between two hops, and node depth of the next hop. This RPSOR protocol results in an increased packet delivery ratio and the end-to-end delay is decreased.

The routing problem is different in the case of multiple gateways. In GCORP (Geographic and Cooperative Opportunistic Routing Protocol) [14], the concept of the multi-sink is introduced. Intermediate relay nodes are placed between source nodes and sink for packet routing. Source nodes determine the relay forwarding set from neighbor relay node based on a depth fitness factor. Weighted scheme is applied on normalized energy, packet delivery probability, and normalize distance. The relay node with highest weight value is selected as best relay node. The protocol results in improved packet delivery ratio, low end-to-end delay, and enhanced network lifetime. However, the protocol suffers from void occurrence and multipath problem.

As already mentioned, the proposed routing algorithm is based on a previous work, the protocol SPRINT [4], which also addresses the energy consumption issue.

## 3. System Model

The scenario considered is a 3D UWSN where nodes are deployed at different depths in an underwater cubic region. A channel model for underwater medium is also implemented considering the environment factors of USWNs that include transmission loss, absorption loss, signal to noise ratio, various noises, and energy consumption during a packet transmission and reception. It is also important to consider the energy consumption parameter while designing protocols for UWSN due to the limited energy available at the nodes.

### 3.1. Network Model

**Figure 1:**
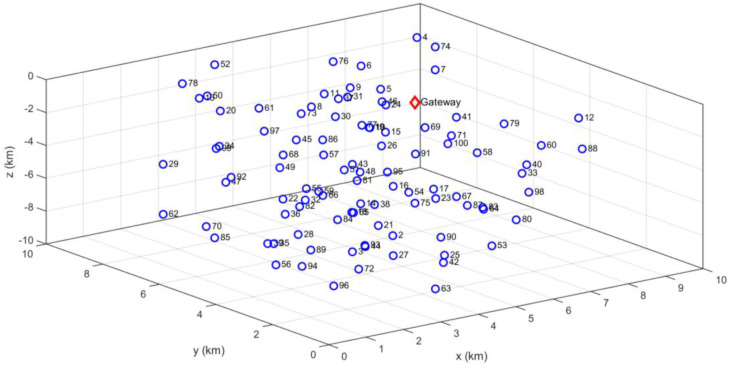
Random deployment of nodes of a 3D underwater wireless sensor network (100 nodes).

The system model is considered three-dimensional with random location of the nodes due to its impact on many important parameters on underwater sensor environment. The surface buoys reside at the water surface and the anchor nodes are connected to these buoys using a rope or cable. In order to place the sensor nodes at intermediate depths, the sensor nodes can be attached to the surface buoy with length adjusted wire or rope. An instance of a possible scenario is shown in Figure 1, where the deployment of nodes is based on random locations including a gateway or sink node that is at the surface. A minimum distance between two nodes is assumed to avoid undesired overlaps.

### 3.2. Propagation Model for Underwater Sensor Networks

**Figure 2:**
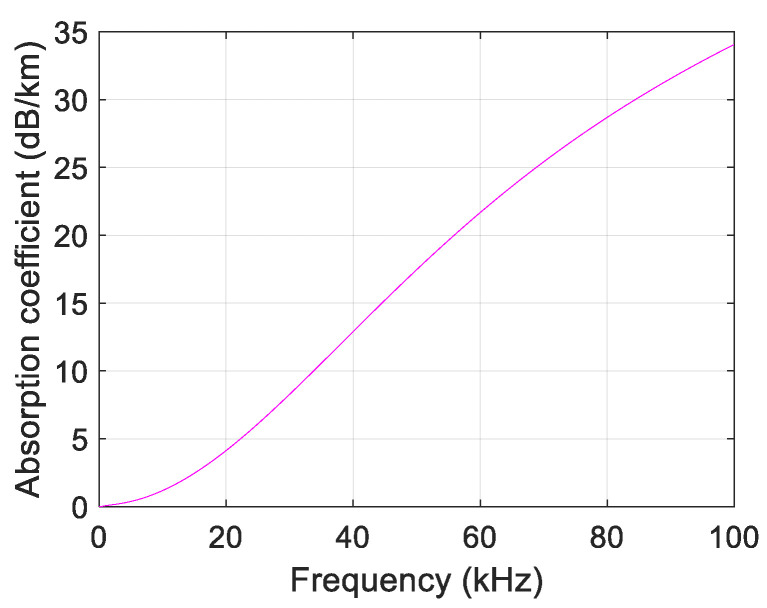
Absorption coefficient vs. frequency.

The submarine channel undergoes with many characteristics including different kinds of noise, multipath fading, a variable propagation speed (in function of salinity, temperature…), and transmission and -absorption loss due to distances. Considering these characteristics, the absorption loss can be expressed as Thorp’s equation [15],

(1) $$\alpha \left(f\right)=\frac{0.11f^{2}}{1+f^{2}}+\frac{44f^{2}}{4100f^{2}}+2.75\;*10^{-4}f^{2}+0.0033\;\left(\frac{\mathrm{dB}}{\mathrm{km}}\right)\;$$

and it is a function of frequency *f*. Equation (1) is an empirical equation that provides a good approximation at frequencies from 100 Hz to 1 MHz. Figure 2 shows the value of (1) up to 100 kHz.

The Transmission Loss (*TL*) is calculated as a function of distance *r* (m) and absorption coefficient α (dB/km). It can be expressed in two ways, in cylindrical ($TL_{CS}$) for shallow waters (depth less than 100 m) and spherical spreading ($TL_{SS}$) for oceanic waters [16], respectively, as

(2) $$TL_{cs}=10\log\left(r\right)+\alpha \left(f\right)*r*10^{-3}$$

(3) $$TL_{ss}=20\log\left(r\right)+\alpha \left(f\right)*r*10^{-3}$$

where *r* indicates the hop distance (m) and *f* is frequency. The speed of sound in underwater is given by *c* (m/s) [17]

(4) $$c\;=1449.2+4.6\;T-0.055\;T^{2}+0.00029\;T^{3}+\left(1.34-0.01\;T\right)\left(S-35\right)+0.016\;d\;,$$

where *T* is temperature in Celsius scale, *S* is salinity in parts per thousand, and *d* is depth in meters. Ambient noise is a contribution of at least four factors: turbulence noise ($N_{t})$, shipping noise $\left(N_{s}\right)$, wave and other surface noise $\left(N_{w}\right)$, and thermal noise $\left(N_{th}\right)$. The frequency dependence of every ambient noise component, is given by [17]

*N*(*f*) = *N*_*t*_ (*f*) + *N*_*s*_ (*f*) + *N*_*w*_ (*f*) + *N*_*t*__*h*_ (*f*), (5)

10 log *N*_*t*_ (*f*) = 17 − 30 log (*f*), (6)

10 log *N*_*s*_(*f*) = 40 + 20 (*s* − 0.5) + 26 log *f* − 60 log (*f* + 0.03), (7)

(8) $$10\;\log\;N_{w}(f)=50+7.5\;\sqrt{w}+20\;\log\;f-40\;\log\;f\;(f+0.4),$$

10 log *N*_*t*__*h*_ (*f*) = −15 + 20 log *f*. (9)

In underwater environment, signal-to-noise ratio (SNR) is based on source level, directivity index, ambient noise, and transmission loss (Equations (2) and (3)). The SNR at the receiver input, can be calculated, in logarithmic scale as [18]

(10) SNR=SL−TL−NL+DL

where *SL* stands for the source level expressed in (dB μPa), which is directly related to the transmitting power; *TL* is the transmission loss (dB); *NL* is the ambient noise; and finally, *DL* is the directivity index (dB) of the transducers.

### 3.3. Model for Energy Consumption

The generalized energy model that is used to calculate the energy needed to deliver a packet (energy/packet) between two nodes separated by distance *d* is given by [19]

(11) $$E_{d}=E_{t}\left(d\right)+E_{r}\left(d\right)$$

where $E_{t}\;$ is the transmission energy/packet and $E_{r}\;$ is the reception energy/packet. Both components can be expressed in terms of other specifics parameters, as seen in the next two equations

(12) $$E_{t}\left(d\right)=L\;\left(E_{elec}+\varepsilon_{amp}\right)+P_{t}\frac{L}{\alpha B\left(d\right)},$$

(13) $$E_{r}\left(d\right)=L\;\left(E_{elec}+E_{DA}\right)+P_{r}\frac{L}{\alpha B\left(d\right)},$$

where *L* is the number of bits in a packet; $P_{t}\;$ and $P_{r}\;$ are the transmission and reception power, respectively, both independent of the distance; $E_{elec}$ is the electronic energy required to process one bit of message; $\varepsilon_{amp}$ is the energy/bit consumed by the amplifier; $E_{DA}\;$ is the energy/bit required for data aggregation; α is the modulation efficiency; and finally *B(d)* is the available bandwidth, which could depend on the distance *d*.

## 4. Proposed Protocol

Considering underwater constraints including the propagation model, harsh environment, water current, and depth in 3D UWSNs, a packet forwarding protocol based on alternate path to conserve the energy is proposed.

The proposed protocol is based on SPRINT protocol [4], which is designed to achieve trade-off between energy consumption and throughput. A packet forwarding node selects one of its neighbors as a relay node. The main criterion to select a relay node is the minimum distance to conserve the energy. The distance is estimated by the received signal strength (RSS). However, minimum distance is not the only criterion. The use of number of hops or relay nodes from source to final destination affects the throughput. Each hop adds to the delay in packet forwarding and, as a result, reduces throughput. Therefore, along with the distance parameter, the number of hops between the relay and the sink and the number of neighbors of the relay node are also taken into consideration. The minimum number of hops is used to maximize the throughput while minimum number of neighbors is used to minimize the traffic congestion and energy consumption of the relay. It is possible that the selected relay node is not the optimal selection due to error in RSS estimation. However, the optimal node may be selected later as the relay selection process is recursive. The routing path formation will be initiated by the sink and data packets will be sent once the routing path formation process is over. To avoid the network overhead and enhance packet delivery ratio, the routing path will be updated recursively at some suitable interval depending on the data packets arrival rate. The distance, energy consumption and number of neighbors are not static parameters of the network. The distance between the two nodes may change due to the limited mobility of the nodes. Furthermore, the energy consumption of some nodes may be higher than the others and number of neighbors may also change because of the nodes limited mobility and failures. As the selection parameters are not static and the optimal routing path is sought, a fuzzy logic scheme to select the relay node has been envisaged. The selection is based on three input parameters: (i) number of hops in the path, (ii) number of neighbors of a node, and (iii) distance from a transmitting node to the forwarding node. In SPRINT protocol, three weight factors are used with those three parameters. In this proposal, a fuzzy inference method is applied to select the forwarding node, and it will be shown that it is possible to reduce both the packet delay and the overall energy used by the network.

**Figure 3:**
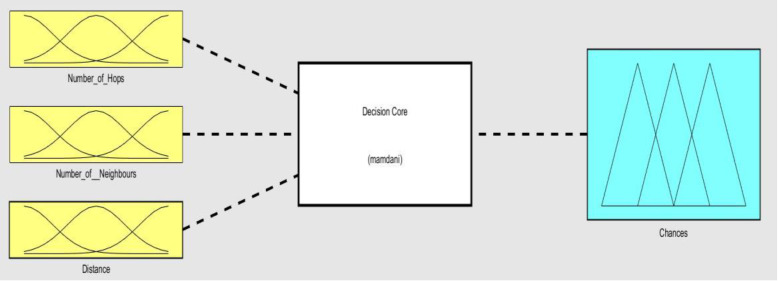
Simplified structure of a fuzzy inference system.

The structure of a fuzzy logic system can be seen in Figure 3. The system has three elements or stages: the input mapping or fuzzification stage, the decision core (also called “fuzzy rules” or “fuzzy logic engine” in technical literature), and the output mapping or defuzzification stage. In the first stage, the so-called membership functions map the possible values of the input variables to the real range [0,1]. Simple analytic canonic functions like triangular, rectangular, or gaussian functions are used as membership functions, although other shapes are also possible, such as sigmoid and bell functions. In this work, triangular functions have been used due to their simplicity. The second stage, the so-called core decision, is implemented by a set of rules (Boolean, *IF-THEN*, …). All rules are evaluated in parallel using fuzzy reasoning. Eventually, the outputs of the core decision stage enter the defuzzification stage, where they are combined to provide a normalized numerical value called Chance, which is the response of the fuzzy logic system. The defuzzification stage also uses a membership function. Fuzzy logic system is described more in detail in the next section. 

### 4.1. Fuzzy Logic

**Figure 4:**
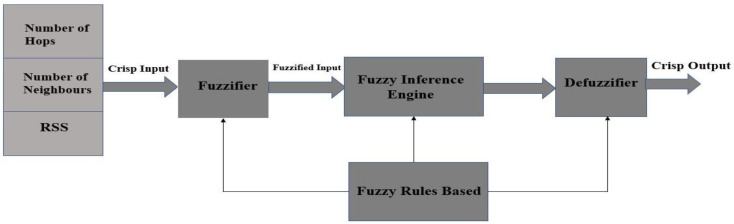
Block diagram of a fuzzy logic system.

Fuzzy logic is a type of multi-valued logic that deals with reasoning to provide an approximate rather than exact result. Fuzzy logic is also used for estimating and making a decision among multiple variables. Figure 4 shows a block diagram that illustrates the block diagram of a fuzzy logic system. Fuzzy inference is the name of the process of mapping a given input to an output using fuzzy logic.

As already mentioned in the previous section, a fuzzy logic system, also called fuzzy inference system, has three stages: Fuzzifier, core decision stage, or Fuzzy Inference Engine, and Defuzzifier, as shown in Figure 4. In the first and third stages, linguistic terms are used to map the stage input variables to the real interval [0,1]. The mapping is performed by the so-called membership functions. The linguistic terms are shown in Table 1 and Table 2. The fuzzy inference provides a basis from which decisions can be made or patterns distinguished [20]. The system output is a real number in the interval [0,1]. The term Chance is used to refer to either the linguistic term or the numerical values. It has been found that Chance is a good name to represent the score of a node to be chosen as forwarding node. The three stages of the system are described below.

(a) Stage 1: Fuzzification

There is a membership function associated to every linguistic term in Table 1. The first stage is to evaluate the membership functions for each input (number of hops, number of neighbors and distance). The triangular membership functions are described as [21]

(14) $$\mu \left(x\right)=\{\begin{array}{c} \begin{array}{cc} 0, & x\;\leq a\\ \frac{x-a}{b-a}, & a\leq x\leq b \end{array}\\ \begin{array}{cc} \frac{c-x}{c-b}, & b\leq x\leq c\\ 0 & c\leq x \end{array} \end{array}$$

**Figure 5:**
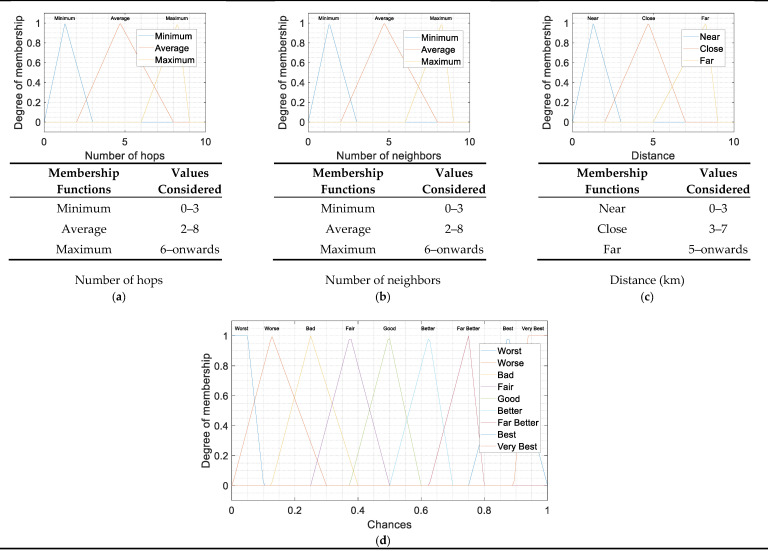
Memberships functions used in the fuzzification (**a**–**c**) and defuzzification (**d**) stages.

The membership function μ(x) provides the degree of membership. In Figure 5a–c, the three used membership functions are shown jointly with the associated linguistics terms.

(b) Stage 2: Fuzzy Rules

Fuzzy rules are based on *IF*-*THEN* consequences by applying Boolean *AND*/*OR* operations to the input. To do that, Mamdani method has been used. As an example, the fuzzy rules for the limit values of Chance are explained (limit values of Chance are Best and Worst; they are used because they are more illustrative than others):

Instance 1: *IF* number of hops are Minimum, *AND* number of neighbors are Minimum *AND* RSSI distance is Near, *THEN* Chance of packet forwarding is Very Best.
Instance 2: *IF* number of hops are Maximum, *AND* number of neighbors are Maximum *AND* RSSI distance is Far, *THEN* Chance of packet forwarding is Worst.

In the problem of underwater routing, the proposal to choose the candidate nodes for packet forwarding is based on the fuzzy rules described in the Table 3.

(c) Stage 3: Defuzzification

The defuzzification stage involves two steps. In the first step, the membership function of Figure 5d is evaluated at the values obtained in the second stage. In the second step, a single number is obtained. In this work, the Center of Mass (CoM) method has been used, and the single number is calculated as

(15) $$z=\frac{\sum_{i=1}^{q}x_{i}\;\mu (x_{i})}{\sum_{i=1}^{q}\mu (x_{i})}\;,$$

where μ(x) are the triangles of Figure 5d, and *z* is Chance. The node with a larger value *z* is the node with better Chance to be the forwarding node. Table 4 shows some examples of the results obtained.

It is well known that using the fuzzy logic to choose the forwarding node becomes very easy compared to SPRINT [4], which uses the weights method using normalized values to select the forwarding node and RECRP [6], which uses RSSI and Doppler scale shift measurement to estimate distance using optimal min-max method, and next hop selection is based on the information in a routing table that is updated from the beginning to the ending node. In [6], due to regular updates of the routing table, the energy consumption will be increased. Similarly, among the neighbors, the forwarding node is chosen based on the largest value. The process is continued until the sink is reached. 

### 4.2. Network Performance

The indicators used to evaluate the proposed scheme have been already introduced: number of hops, number of neighbors, and RSSI. Additionally, different transmission ranges have been considered to assess the performance of the system in terms of energy consumption and average number of hops. The energy consumption in UWSNs is usually due to network operations such as processing, gathering, forwarding, and receiving data. Therefore, the total energy consumption is the energy dissipated due to these actions in the nodes.

Number of hops refers to intermediate nodes that a packet must visit to reach the destination which is the gateway. This parameter has a direct relationship with the distance, which is the third indicator, obtained from the RSSI. Distance is related to energy consumption. Due to the law of transmission power proportional to the square of the distance, multi-hop communication is preferred. Nevertheless, the energy used in a node for receiving and processing a message, and not for transmission, might modify this criterion. However, the larger the number of hops, the larger the end-to-end delay.

Number of neighbors is the second indicator the select the forwarding node. This indicator is a measure of the priority of selecting the forwarding node. The lower number of neighbors implies a greater chance of a node to be selected as forwarding node.

## 5. Simulation Results and Discussion

The energy performance of the protocol is analyzed in terms of two magnitudes: average and total energy consumption by nodes. The algorithms and protocols described in Section 4 have been developed in MATLAB^®^. For the simulations, a variable number of nodes, up to 600, have been quasi-randomly located in the scenario, which is a cubic region of side 10 km. Here, quasi means that there is the restriction of a minimum distance between nodes; they that cannot be within 1 km of each other to avoid undesired overlaps. The speed of sound could be calculated with Equation (4). In the simulations, the used value is 1500 m/s. Several transmission ranges have been considered, from 1 km to 8 km. Ten (10) cases were simulated for each value of the transmission range. The detailed summary of parameters used is given in Table 5.

MATLAB^®^ was chosen to implement the protocol. Three average figures vs. transmission range were calculated: average energy consumed per node and per packet (energy/node/packet), average number of hops, and average total energy.

**Figure 6:**
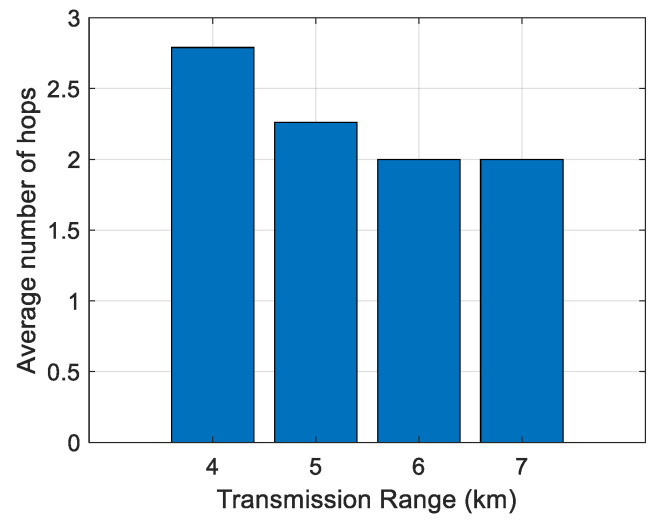
Average number of hops for 100 nodes.

**Figure 7:**
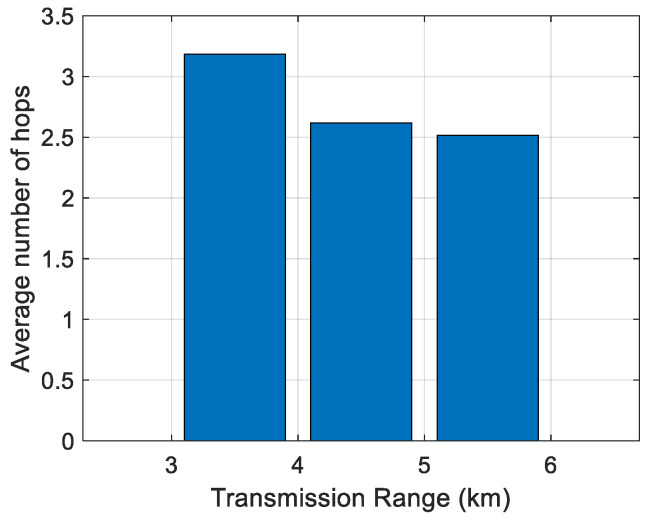
Average number of hops for 175 nodes.

**Figure 8:**
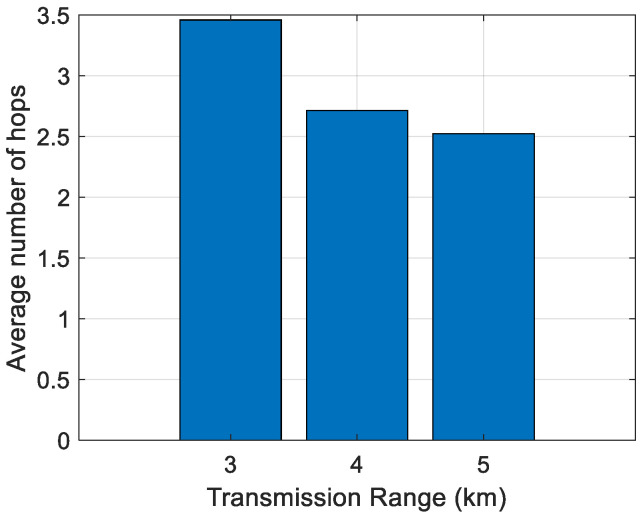
Average number of hops for 250 nodes.

**Figure 9:**
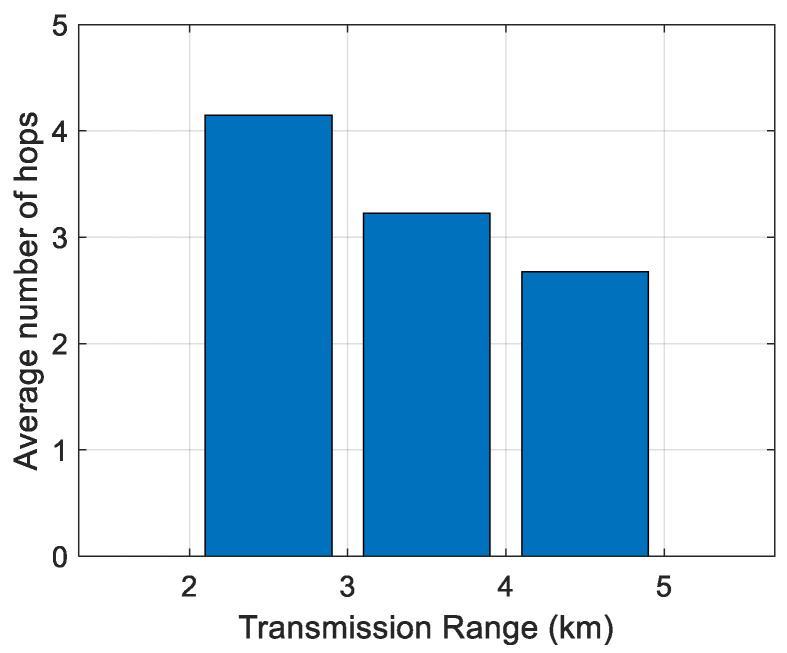
Average number of hops for 325 nodes.

**Figure 10:**
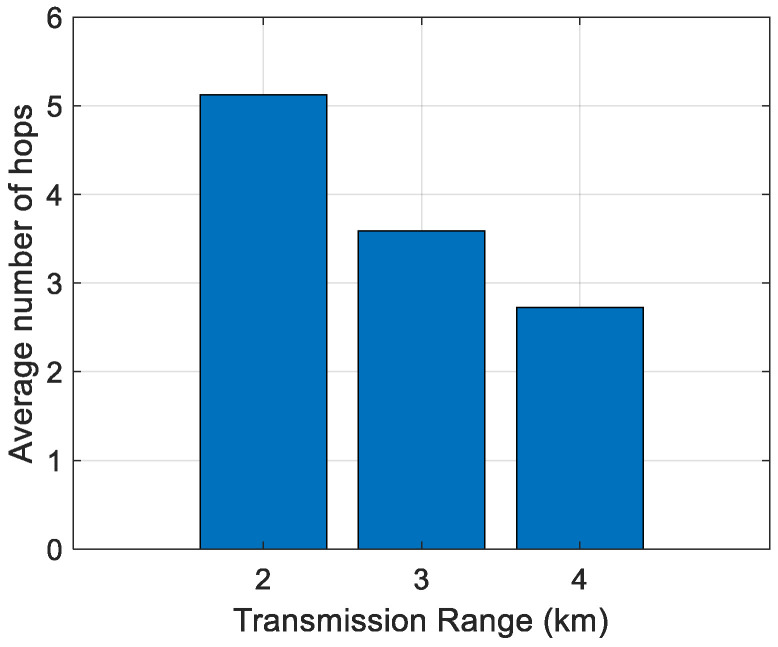
Average number of hops for 400 nodes.

First, the impact of the transmission range on the average number of hops has been analyzed. The results are shown in Figure 6, Figure 7, Figure 8, Figure 9 and Figure 10. It can be observed that average number of hops decreases with increasing transmission range.

As mentioned at the end of the previous section concerning the law of transmission power proportional to the square of the distance, the multi-hop scheme could be more efficient. In addition, and not least, the end-to-end delay increases with the number of hops. For these two reasons, a path with fewer jumps is preferred.

Simulations were also carried out to analyze the influence of the transmission range on the energy/node/packet and the average number of hops. When there are fewer nodes in the scenario, the transmission range must be longer, and the opposite. The transmission ranges considered for the different number of nodes are shown in Table 6. The simulation results of the average number of hops vs. transmission range are shown in Figure 6, Figure 7, Figure 8, Figure 9 and Figure 10.

**Figure 11:**
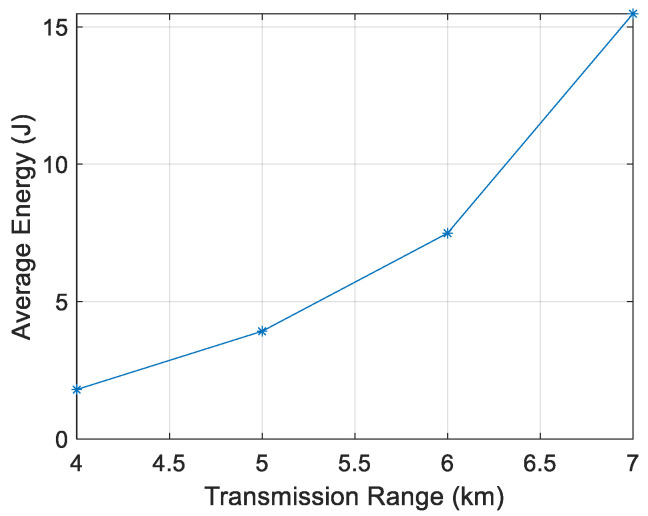
Energy/node/packet for 100 nodes.

**Figure 12:**
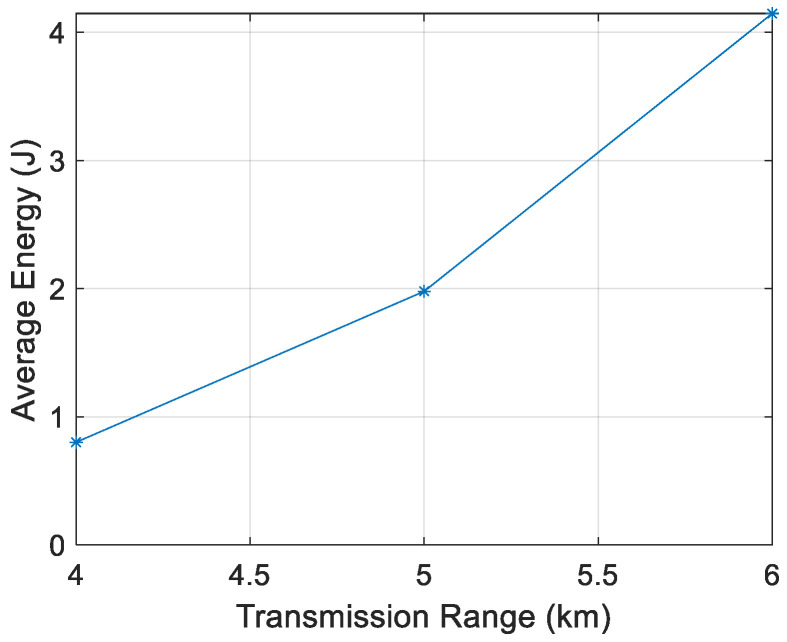
Energy/node/packet for 200 nodes.

**Figure 13:**
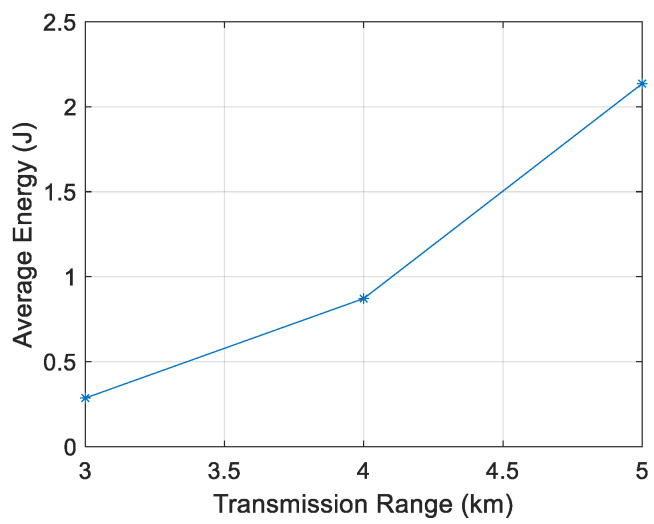
Energy/node/packet for 300 nodes.

**Figure 14:**
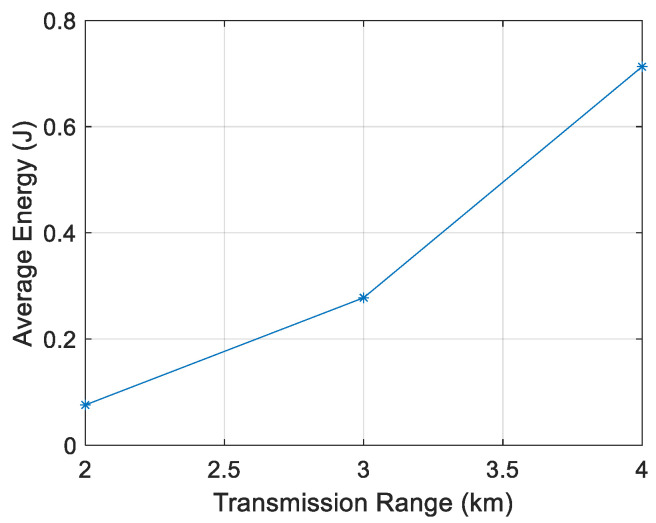
Energy/node/packet for 400 nodes.

Figure 11, Figure 12, Figure 13 and Figure 14 show that the energy/node/packet increases with the transmission range, as described above. It can also be observed that the energy decreases with the node density. For instance, with a transmission range of 4 km, the energy/node/packet is 1.9 J with 100 nodes and goes down to 0.71 J with 400 nodes. 

**Figure 15:**
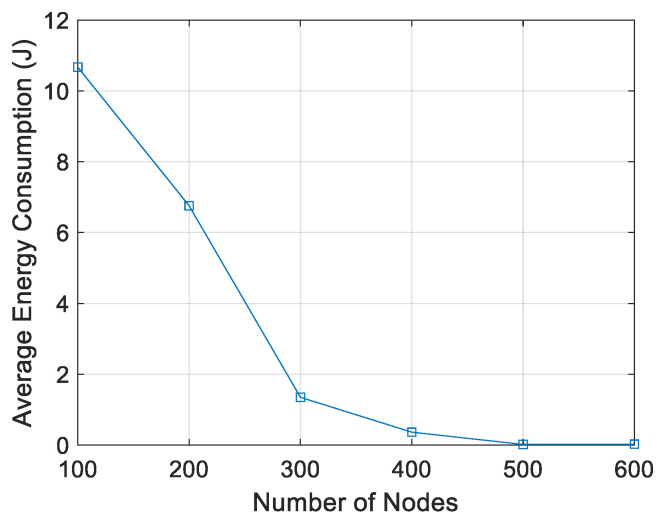
Energy/node/packet vs. number of nodes.

As shown in Figure 15, the larger number of nodes, the lower energy/node/packet. When there are 100 nodes, the energy used is approximately 10.9 J and for 600 nodes it is ~0.2 J. It can be seen that a higher node density results in lower energy/node/packet.

**Figure 16:**
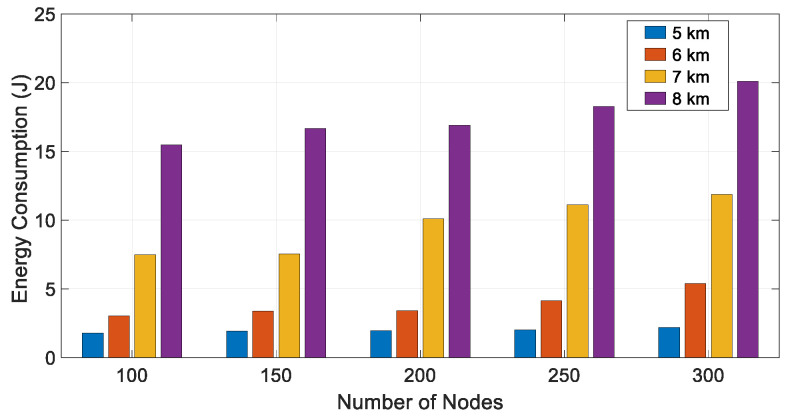
Energy/node/packet used vs. number of nodes for different transmission ranges.

Figure 16 shows the energy/node/packet used vs. the number of nodes, with transmission range as parameter. There is not a clear trend of average consumption vs. number of nodes, but it clearly increases with the transmission range. A good observation from Figure 16 is that, in terms of energy consumption, the network is scalable and stable, that is, the energy used does not show abrupt increments with the network size.

**Figure 17:**
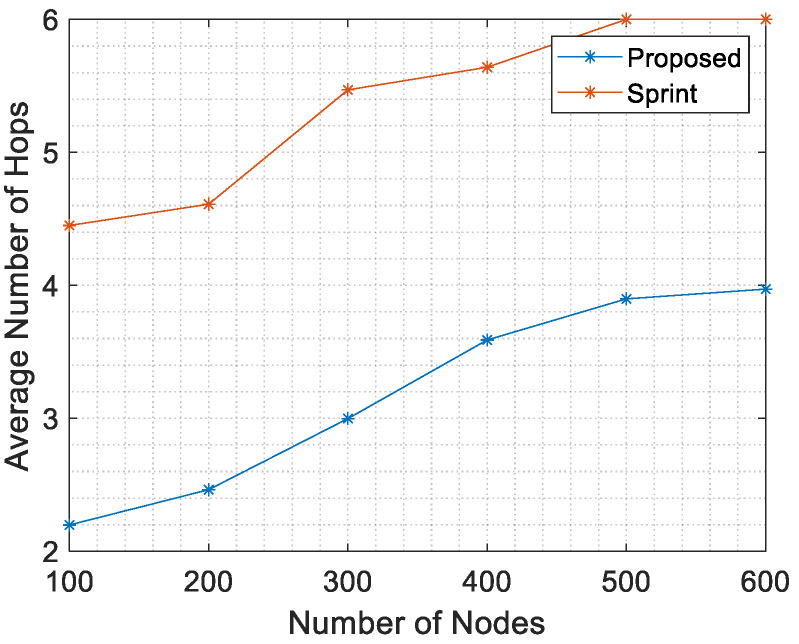
Average number of hops vs. number of nodes.

The effect of network size (actually, the network density) on the average number of hops is shown in Figure 17, jointly with the average number of hops of SPRINT protocol taken from [4]. In comparison of two graphs, it is well evident that average number of hops of the proposed fuzzy scheme is lower than the same figure obtained with SPRINT protocol. 

**Figure 18:**
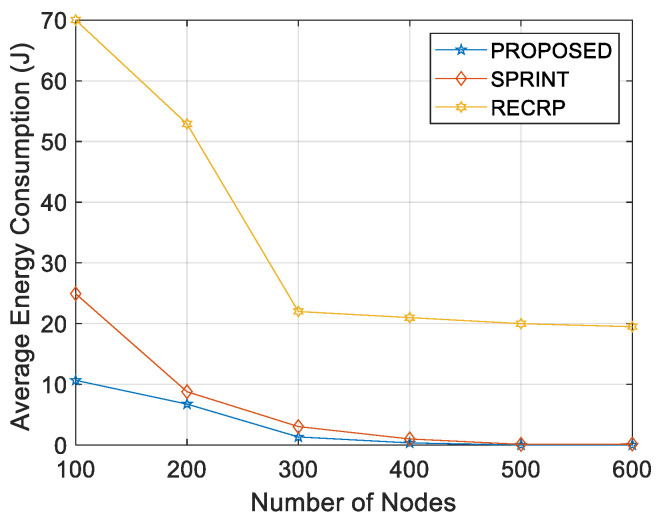
Energy/node/packet vs. number of nodes.

Figure 17 and Figure 18 show a comparison of the results of the proposed protocol and the same results of the SPRINT protocol. The fuzzy inference scheme finds paths with fewer hops and lower energy consumption. Table 7 shows a comparison in terms of energy consumption between the proposed protocol and both SPRINT and RECRP [6].

## 6. Discussion

UWSNs suffer from limited energy available to operate. The routing scheme is of paramount importance in that scenario. A smart path selection can also improve other performance indicators of the network, as the end-to-end-delay. In this paper, a forwarding node selection algorithm has been proposed, based on fuzzy inference, for the SPRINT protocol.

The objective of the work was to improve the energy efficiency of an UWSN routing protocol, which also helped to reduce the number of hops and, consequently, the end-to-end delay. The contributions of the work are (i) the use of fuzzy inference to select the forwarding node to form the path, (ii) the set of rules that form the logic of the fuzzy inference, and (iii) the effect of the transmission range on the number of hops to reach the gateway and on the average energy consumption.

The fuzzy inference has been implemented in MATLAB^®^. The input variables to the fuzzy logic algorithm are distance (through RSSI value), number of neighbors, and number of hops.

Simulations were carried out to obtain the energy/node/packet and number of hops vs. transmission range and number of nodes. In fact, number of nodes means node density, because the considered scenario has a fixed volume.

If the transmission circuit is the main energy consumer, it seems that less hops need more power, due to the transmission power law proportional to the squared distance. However, the simulations show that it is possible to find a path with fewer hops, but still less energy consumption. Moreover, fewer hops mean shorter end-to-end delays.

The algorithm improves the efficiency of the USWN in terms of used energy and number of hops, which also reduces the end-to-end delay. Simulations results of the proposed scheme show a more energy efficient performance when compared to other UWSN routing protocols as SPRINT and RECRP.

## Figures and Tables

**Table 1:** Fuzzification linguistic terms.

Input	Membership		
Number of hops	Minimum	Average	Maximum
Number of neighbors	Minimum	Average	Maximum
Distance	Near	Close	Far

**Table 2:** Defuzzification linguistic terms.

	Linguistic Variables
Chance	Very Best, Best, Far Better, Better, Good, Fair, Bad, Worse, Worst

**Table 3:** Fuzzy rules established for the proposed scheme.

No.	No.	No.	Distance	Chance	No.	No.	No.	Distance	Chance
Rule	Hops	Neighbors			Rule	Hops	Neighbors		
**1**	Min.	Min.	Near	Very Best	15	Max.	Avg.	Near	Good
**2**	Min.	Min.	Close	Best	16	Min.	Avg.	Near	Good
**3**	Max.	Min.	Close	Best	17	Avg.	Min.	Near	Good
**4**	Min.	Avg.	Close	Best	18	Min.	Avg.	Far	Good
**5**	Max.	Min.	Close	Best	19	Min.	Min.	Far	Good
**6**	Max.	Max.	Close	Far Better	20	Max.	Min.	Near	Good
**7**	Max.	Avg.	Close	Far Better	21	Avg.	Avg.	Far	Fair
**8**	Min.	Min.	Far	Far Better	22	Avg.	Max.	Near	Fair
**9**	Min.	Max.	Close	Better	23	Avg.	Max.	Far	Bad
**10**	Avg.	Max.	Close	Better	24	Max.	Max.	Far	Bad
**11**	Avg.	Avg.	Close	Better	25	Max.	Max.	Near	Bad
**12**	Avg.	Min.	Near	Better	26	Avg.	Max.	Far	Worse
**13**	Avg.	Avg.	Near	Better	27	Max.	Max.	Far	Worst
**14**	Avg.	Min.	Far	Better					

Note: Min. = minimum, Med. = medium, Max. = maximum, Avg. = average.

**Table 4:** Result of fuzzy operation.

Case	No.	No.	Distance	Chance
No.	Hops	Neighbors		
1	7	6	8	0.375
2	6	6	6	0.474
3	5	8	6	0.312
4	3	4	5	0.625
5	7	3	2	0.564
6	2	3	4	0.875
7	1	2	7	0.637
8	5	8	7	0.196
9	1	1	1	0.929
10	1	1	2	0.917

**Table 5:** MATLAB^®^ simulation parameters.

Simulation Parameters	Value	Unit
Speed of sound	1500	m/s
Data rate	5000	bit/s
Frequency	48	kHz
Packet length	256	bit
Header length	30	bit
Transmission power	18	W
Number of nodes	100 to 600	-
Simulation length	10	-
Minimum distance	1	km
Transmission range	2 to 8	km

**Table 6:** Transmission ranges and number of nodes.

Number of Nodes	Transmission Range (km)
100	4, 5, 6, 7
175	3.5, 4.5, 5.5
250	3, 4, 5
325	2.5, 3.5, 4.5
400	2, 3, 4

**Table 7:** Energy consumption comparison.

Energy (J)			
Number of Nodes	Proposed	SPRINT	RECRP
100	10.5	25.56	70
200	6.55	8.451	52.9
300	3.10	3.433	22
400	0.99	1.249	21
500	0.25	0.528	20
600	0.15	0.637	19.5

## Data Availability

Not applicable.
